# Bactericidal activity of M protein conserved region antibodies against group A streptococcal isolates from the Northern Thai population

**DOI:** 10.1186/1471-2180-6-71

**Published:** 2006-08-09

**Authors:** Nonglak Yoonim, Colleen Olive, Chulabhorn Pruksachatkunakorn, Sumalee Pruksakorn

**Affiliations:** 1 Department of Microbiology, Faculty of Medicine, Chiang Mai University, Chiang Mai 50200, Thailand; 2 Department of Pediatrics, Faculty of Medicine, Chiang Mai University, Chiang Mai 50200, Thailand; 3 Queensland Institute of Medical Research, 300 Herston Road, Herston, Brisbane, QLD 4006, Australia

## Abstract

**Background:**

Most group A streptococcal (GAS) vaccine strategies have focused on the surface M protein, a major virulence factor of GAS. The amino-terminus of the M protein elicits antibodies, that are both opsonic and protective, but which are type specific. J14, a chimeric peptide that contains 14 amino acids from the M protein conserved C-region at the carboxy-terminus, offers the possibility of a vaccine which will elicit protective opsonic antibodies against multiple different GAS strains. In this study, we searched for J14 and J14-like sequences and the number of their repeats in the C-region of the M protein from GAS strains isolated from the Northern Thai population. Then, we examined the bactericidal activity of J14, J14.1, J14-R1 and J14-R2 antisera against multiple Thai GAS strains.

**Results:**

The *emm *genes of GAS isolates were sequenced and grouped as 14 different J14-types. The most diversity of J14-types was found in the C1-repeat. The J14.1 type was the major sequence in the C2 and C3-repeats. We have shown that antisera raised against the M protein conserved C-repeat region peptides, J14, J14.1, J14-R1 and J14-R2, commonly found in GAS isolates from the Northern Thai population, are able to kill GAS of multiple different *emm *types derived from an endemic area. The mean percent of bactericidal activities for all J14 and J14-like peptide antisera against GAS isolates were more than 70%. The mean percent of bactericidal activity was highest for J14 antisera followed by J14-R2, J14.1 and J14-R1 antisera.

**Conclusion:**

Our study demonstrated that antisera raised against the M protein conserved C-repeat region are able to kill multiple different strains of GAS isolated from the Northern Thai population. Therefore, the four conserved "J14" peptides have the potential to be used as GAS vaccine candidates to prevent streptococcal infections in an endemic area.

## Background

*Streptococcus pyogenes *or group A streptococcus (GAS) is a human bacterial pathogen that colonizes the throat or skin surfaces of the host. GAS infection can lead to a number of diseases including pharyngitis, impetigo and necrotising fasciitis. In a small percentage of individuals that are left untreated or are treated ineffectively with antibiotics, streptococcal infections can lead to more serious illnesses such as rheumatic fever (RF) and rheumatic heart disease (RHD) which are a significant health concern in developing countries [1].

Most GAS vaccine strategies have focused on the M protein, a major virulence factor of GAS. The M protein has an alpha helical coiled-coil structure comprised of a variable amino terminal domain followed by a set of three repeat regions called A, B and C-repeats, a cell wall anchor motif and a stretch of hydrophobic amino acids which are embedded in the cell membrane. Antibodies to the highly variable amino terminal region of the M protein have been shown to be opsonic and protective in murine models and correlate with protection in humans [2-5]. However, there are more than 150 recognized *emm *genotypes [6] and an increasing number of non-M typeable strains [7-9]. Therefore, type-specific antibodies are ineffective in providing broad-spectrum protection against multiple different GAS strains. Strategies employed to develop a broad strain coverage GAS vaccine have included the design of multivalent constructs containing type-specific M protein sequences [10-13] associated with a particular disease or geographical region and the identification of vaccine candidates based on the conserved C-region of the M protein [14-17].

Many studies have investigated the potential of the M protein C-repeat region that is conserved among different GAS strains as a vaccine candidate [14-17]. Using a series of 15 overlapping peptides spanning the entire M protein C-region, a peptide LRRDLDASREAKKQVEKALE (p145) that is recognized by antibodies in the sera of most adults living in areas of high GAS exposure was identified [16,18]. The acquisition of these antibodies with age paralleled the acquisition of GAS immunity indicating the potential use of p145 as a vaccine candidate. Human sera with antibodies to p145 have also been shown to be opsonic against heterologus GAS strains. Similarly, mice immunized with p145 elicited antibodies that were opsonic against GAS [2,3]. However, several studies indicated that p145 contained a T cell epitope shared with determinants on human cardiac myosin, and keratin in mouse [19]. In another study [20], J14 (KQAEDKVK**ASREAKKQVEKALE**QLEDRVK), a peptide with minimal B and T cell epitopes within p145 was identified as a GAS M protein C-region peptide devoid of potentially deleterious T cell autoepitopes, but which contained an opsonic B cell epitope. J14 offers the possibility of a vaccine which will elicit protective opsonic antibodies against multiple different GAS strains. J14 is a chimeric peptide that contains 14 amino acids from M protein C-region (shown in bold) and is flanked by yeast-derived GCN4 sequences which was necessary to maintain the correct helical folding and conformational structure of the peptide.

From GenBank database search and many studies [3,21], there are about 60% of GAS that contain J14 sequences, while the remaining contain J14-like sequences. Although J14 has the potential to be a vaccine candidate to prevent streptococcal infection, there are still one-third of M types that do not contain the J14 sequence but contain J14-like sequences. This study utilised three peptides, KQAEDKVK**ASREAKKKVEADLA**QLEDRVK (J14.1), KQAEDKVK**ASREAKKQVEKDLA**QAEDKVK (J14-R1) and KQAEDKVK**ASRAAKKELEAEHQ**QAEDKVK (J14-R2), representing commonly occurring J14-like sequences, in addition to J14, and assessed their ability to elicit broadly opsonic antibodies following immunization of mice, and potential as vaccine candidates. It is likely that a vaccine incorporating J14 and J14-like sequences would be more beneficial in providing protection against streptococcal infections covering the majority of GAS M types.

## Results and discussion

We searched for J14 and J14-like sequences and the number of their repeats in the C-terminal region of the M protein from GAS strains isolated from the Northern Thai population. Then, we examined the bactericidal activity of J14, J14.1, J14-R1 and J14-R2 antisera against multiple Thai GAS strains. This data is important to the development of an appropriate vaccine against GAS infection in a specific endemic population.

### Distribution of J14 and J14-like sequences

Twenty different *emm *types of GAS isolated from ChiangMai, Thailand were included in this study. *Emm *types were analyzed by sequencing the N-terminal region of *emm *genes. The C-repeat region of *emm *genes of these isolates were sequenced to examine the distribution of J14 and J14-like sequences. The majority of the isolates 12/20 (60%) contained three C-repeats. Seven of 20 isolates (35%) contained two C-repeats. Only one isolate (5%), ST9, contained a single C-repeat (Table 1).

We found 14 different J14-types which were J14, J14.1, J14-R1 to J14-R12. The most diversity of J14-types was found in the C1-repeat which contained 9 different J14-types (Table 1). The C2-repeat contained 8 different J14-types with the majority being J14.1 (Table 1). In addition, J14.1 is also the major sequence within the C3-repeat. J14 was only found in the C3-repeat.

Although J14 has the potential to be a vaccine candidate to prevent streptococcal infection, only 5 of 20 (25%) GAS isolates in our study contained the J14 sequence in the C-repeat region. Interestingly, the J14.1 sequence was found with a higher rate, 12 of 20 (60%), than J14. In addition, we found that J14-R1 and J14-R2 are common among the remaining J14-types. Therefore, the four "J14" peptides; J14, J14.1, J14-R1 and J14-R2, are promising GAS vaccine candidates.

### Bactericidal activity of GAS isolates

We have examined the bactericidal activity (% reduction in CFU) of J14, J14.1, J14-R1 and J14-R2 antisera against GAS isolates from Chiang Mai, Thailand.

Strong bactericidal activity (>70% reduction) was found against 75% (15/20), 80% (16/20), 65% (13/20) and 75% (15/20) of GAS isolates when tested with J14, J14.1, J14-R1 and J14-R2 antisera, respectively (Table 2). Medium bactericidal activity (50-70% reduction) was found against 25% (5/20), 15% (3/20), and 10% (2/20) of GAS isolates when tested with J14, J14-R1 and J14-R2 antisera, respectively (Table 2). Low bactericidal activity (<50% reduction) was found against 20% (4/20), 20% (4/20), and 15% (3/20) of GAS isolates when tested with J14.1, J14-R1 and J14-R2 antisera, respectively (Table 2).

We also compared bactericidal activity of peptide antisera against GAS containing different numbers of C-repeats. The mean percent reduction in CFU of isolates with a single C-repeat was significantly lower than isolates with two or three C-repeats (Table 3). This result corresponded to a study of Vohra *et al *[21] which found that the mean percent reduction in CFU of isolates with two C-repeats was statistically lower than strains with three C-repeats.

Recently, Sandin *et al*. [22] and McArthur *et al*. [23] have commented on the capacity of fibrinogen and albumin to bind to the B- and C-repeats, respectively, causing inhibition of antibody binding under physiological conditions. Nevertheless, several studies [18,21,24-26] demonstrated the binding of anti-C-repeat antibody to GAS isolates, which are in agreement with our study.

Then, we examined the correlation between the opsonization specificity of peptide antisera and J14-types. We found no correlation between the opsonization ability of peptide antisera and J14-types contained in the C-repeats. For example, all J14 peptide antisera showed strong bactericidal activity (>70% reduction) against isolates 2 and 3 having only J14.1 with two C-repeats and isolate 9 also having only J14.1 but with three C-repeats.

These data indicate that these four peptides, J14, J14.1, J14-R1 and J14-R2, have the potential to be used as GAS vaccine candidates to prevent streptococcal infections.

For further study towards the development of a broad strain protective GAS vaccine, it may be possible to use a strategy designed to assemble all four J14 peptides into a single construct [27]. This would have several advantages, including inducing a heterologous opsonic immune response and also providing protection against challenge with many different GAS strains [4]. One such technology is the lipid core peptide (LCP) technology [25,28] which has been used to incorporate up to four different peptides into a single LCP vaccine construct [29]. This system also incorporates the carrier and adjuvant into the vaccine and has the potential for the development of self-adjuvanting multi-antigen component vaccines for human application [25,30].

## Conclusion

Our study demonstrated that antisera raised against the M protein conserved C-repeat region are able to kill multiple different GAS strains isolated from the Northern Thai population. Therefore, the four conserved "J14" peptides have the potential to be used as GAS vaccine candidates to prevent streptococcal infections in an endemic area.

## Methods

### Bacteria

GAS strains used in this study were isolated from the normal population and patients with sore throat, rheumatic heart disease or impetigo in 1985, 1990, 1995, and 2000 from Chiang Mai, Thailand.

### DNA isolation and PCR

DNA was isolated from GAS based on the method previously described [8,31]. The sense primer (CAGTATTCGCTTAGAAAATT AAAA) is derived from the conserved leader sequence of the *emm *gene [32]. The antisense primer for OF-positive *emm *gene, P49 (TTGGGATCCTGCTGATCTT GAACGGTTAGC), is derived from the conserved region of the membrane anchor [33]. The antisense primer for OF-negative *emm *gene, P6 (TGCGGATCCAGCTGTT GCCATAACAGTAAG), is derived from the conserved region of the proline/glycine rich region [33]. The PCR conditions were 94°C for 1 minute, 35 cycles at 94°C for 30 second, 45°C for 30 second, and 72°C for 2 minutes, ending with 72°C for 10 minutes.

### Searching for J14 and J14-like sequences and the number of their repeats in the conserved carboxy terminal segment of the M protein

The PCR products of *emm *gene were sequenced using the ABI Dye Terminator Cycle Sequencing Ready Reaction Kit following the manufacturer's instructions (The Perkin-Elmer Corporation) and determined by an ABI 310 automated sequencer (The Perkin-Elmer Corporation). The obtained DNA sequences of the conserved carboxy terminal segment of the M protein were deduced into an amino acid sequence and then searched for J14 sequence or J14-like sequences. The number of their repeats was recorded.

### Peptides

J14, J14.1, J14-R1 and J14-R2, containing 29 amino acid sequences KQAEDKVK**ASREAKKQVEKALE**QLEDRVK, KQAEDKVK**ASREAKKKVEADLA**QLEDRVK, KQAEDKVK**ASREAKKQVEKDLA**QAEDKVK and KQAEDK VK**ASRAAKKELEAEHQ**QAEDKVK, respectively, were synthesized by the "tea-bag" method. Purity was checked by HPLC. Peptides were dissolved in water at a concentration of 10 mg/ml and kept at -20°C until used.

### Antisera

Antisera to J14, J14.1, J14-R1 and J14-R2 peptides were raised in B10.BR mice. Mice, eight per group, were immunized subcutaneously at the tail base with 30 μg of peptide emulsified in complete Freund's adjuvant (a total of 50 μl was administered). Mice were given subsequent booster injections at intervals of 7 days with 3 μg peptide dissolved in PBS for 5 boosts. Prior to boosting, mice were bled and sera isolated were kept at -20°C until used. Antibody production was assessed by ELISA. Sera showing titer more than 6400 were pooled and used in indirect bactericidal assays to assess their ability to opsonize GAS.

### ELISA

Peptides were diluted to 5 μg/ml in carbonate-bicarbonate buffer, pH 9.6, and coated onto microtiter plates in a volume of 100 μl per well overnight at 4°C. Excess antigen was removed and the wells were blocked with 200 μl of 5% skim milk in PBS-Tween 20 for 1.5 hr at 37°C. Plates were washed five times with PBS-Tween 20. Serum dilutions prepared in 0.5% skim milk in PBS-Tween 20 were added and incubated for 1.5 hr at 37°C. Plates were washed five times with PBS-Tween 20 and incubated with peroxidase conjugated sheep-anti-mouse IgG at a dilution of 1:3000 in 0.5% skimmed milk for 1.5 hr at 37°C. Plates were washed five times with PBS-Tween 20. 100 μl of *o*-phenylenediamine (OPD) substrate was added and incubated for 30 min. The optical density was measured at 450 nm in an ELISA plate reader. The highest dilution that gave an O.D. 3× higher than those of the average of control wells containing normal mouse serum at the same dilution was defined as the titer.

### Indirect bactericidal assay

GAS isolates were cultured overnight in Todd-Hewitt broth at 37°C and diluted to 10^-5 ^dilution in sterile NSS and kept on ice. Fifty μl of the bacterial dilution were plated out in duplicate using the pour plate method and incubated at 37°C for 24 hrs. The numbers of colonies were counted and the colony forming units (CFUs) from these plates were determined as the inoculum size. Fifty μl of the bacterial dilution were also mixed with 50 μl of serum (normal mouse or immune mouse) and then 400 μl of non-opsonic heparinized human donor blood was added. The mixtures were incubated end-over-end at 37°C for 3 hrs, and then 50 μl were plated out in duplicate using the pour plate method. CFUs were counted after 24 hrs of incubation. Bactericidal activity of immune sera (% reduction in mean CFU) was calculated as: 1-[mean CFUs in the presence of immune mouse sera/mean CFUs in the presence of normal mouse sera] × 100. Blood and bacterial controls were included with each isolate of GAS. Each isolate of GAS was done in quadruplicate.

## Authors' contributions

NY carried out the microbiologic experiments, performed the molecular genetic analysis, participated in the sequence alignment, interpretation of data and drafted the manuscript. CO contributed to the editing of the manuscript and partly supervised the study as a collaborator. CP provided clinical specimens and clinical support. SP conceived, designed and supervised the study. All authors read and approved the final manuscript.

## Figures and Tables

**Table 1:** Sequence of J14 types in the M protein C-repeat region of Thai GAS isolates

**Isolate no.**	emm type*	**Year of collection**	**Source of isolation**	**C1-repeat**	**C2-repeat**	**C3-repeat**
1	ST9	1985	throat	-	-	J14

2	M109	1990	nares	-	J14.1	J14.1
3	M70	1985	throat	-	J14.1	J14.1
4	M44	2000	skin	-	J14-R2	J14.1
5	M66	1985	throat	-	J14-R5	J14.1
6	M3	-	Ref	-	J14-R1	J14
7	ST1	1985	throat	-	J14-R9	J14-R6
8	STBSA29	2000	skin	-	J14-R4	J14-R11

9	M49	1985	skin	J14.1	J14.1	J14.1
10	M22	1985	throat	J14-R2	J14.1	J14.1
11	ST8	2000	hand	J14-R4	J14.1	J14.1
12	M75	1985	skin	J14-R6	J14.1	J14.1
13	TR2612	2000	blood	J14-R7	J14.1	J14.1
14	ST3	2000	skin	J14-R10	J14.1	J14.1
15	ST10	1995	throat	J14-R3	J14-R1	J14.1
16	ST6735	2000	leg	J14-R5	J14.1	J14-R12
17	M13	-	Ref	J14-R2	J14-R2	J14-R2
18	M18	-	Ref	J14-R1	J14-R1	J14
19	M74	1985	throat	J14-R3	J14-R3	J14
20	M5	-	Ref	J14-R3	J14-R8	J14

* *emm *gene sequencing. Ref, Reference strain from Health Protection Agency Center for Infection, Respiratory and Systemic Infection Laboratory, London, UK

J14, ASREAKKQVEKALE. J14.1, ASREAKKKVEADLA. J14-R1, ASREAKKQVEKDLA. J14-R2, ASRAAKKELEAEHQ. J14-R3, ASREAKKQLEAEHQ. J14-R4, ASRAAKKELEAKHQ. J14-R5, ASRAAKKDLEAEHQ. J14-R6, ASRAAKKELEANHQ. J14-R7, GSRAAKKELEAKHQ. J14-R8, ASREAKKQLEAEQQ. J14-R9, ASREAKKELEANHQ. J14-R10 ASRAAKKGLEAEHQ. J14-R11, ASREANKKVTSELT. J14-R12, ASRAAKKKVEADLA.

**Table 2:** Bactericidal activity* (% reduction in CFU) of J14 peptide antisera against Thai GAS isolates with different *emm *types

**Isolate no.**	***emm *type**	**J14 (KALE)**		**J14.1 (ADLA)**		**J14-R1 (KDLA)**		**J14-R2 (AEHQ)**	
		
		**IMS/NMS**	**% reduction**	**IMS/NMS**	**% reduction**	**IMS/NMS**	**% reduction**	**IMS/NMS**	**% reduction**
1	ST9	94.5 ± 20.8/200.6 ± 81.8	52.89 ± 10.36	110.5 ± 19.3/200.6 ± 81.8	44.92 ± 9.62	123.5 ± 17.3/200.6 ± 81.8	38.43 ± 8.63	82.9 ± 17.1/200.6 ± 81.8	58.67 ± 8.54

2	M109	34.6 ± 9.5/4025 ± 1753	99.14 ± 0.24	45.1 ± 28.6/4025 ± 1753	98.88 ± 0.71	38.3 ± 26.4/4025 ± 1753	99.05 ± 0.66	25.6 ± 7.8/4025 ± 1753	99.36 ± 0.19
3	M70	215.5 ± 136.5/5171 ± 1916	95.83 ± 2.64	123.9 ± 101.9/5171 ± 1916	97.60 ± 1.97	217.1 ± 112.9/5171 ± 1916	95.80 ± 2.18	152.5 ± 55.2/5171 ± 1916	97.05 ± 1.07
4	M44	10.5 ± 6.4/297.6 ± 140	96.47 ± 2.16	14.1 ± 7.5/297.6 ± 140	95.25 ± 2.52	63 ± 22.7/297.6 ± 140	78.83 ± 7.61	3.8 ± 3.1/297.6 ± 140	98.72 ± 1.03
5	M66	31.8 ± 9.5/117 ± 129	72.86 ± 8.11	11.2 ± 8.3/117 ± 129	90.46 ± 7.05	18.7 ± 11.1/117 ± 129	84.05 ± 9.45	5.2 ± 2.6/117 ± 129	95.58 ± 2.19
6	M3	102.9 ± 43.8/1514 ± 532	93.21 ± 2.89	268.4 ± 69.7/1514 ± 532	82.28 ± 4.60	634.3 ± 187.7/1514 ± 532	58.11 ± 12.4	30.4 ± 11.4/1514 ± 532	97.99 ± 0.75
7	ST1	7.5 ± 2.3/20.8 ± 8.2	63.94 ± 11.02	4.1 ± 1.5/20.8 ± 8.2	80.17 ± 7.18	10.2 ± 2.8/20.8 ± 8.2	51.12 ± 13.24	11.3 ± 1.3/20.8 ± 8.2	45.51 ± 6.05
8	STBSA29	16.4 ± 9.7/59.9 ± 44.9	72.66 ± 16.16	15.0 ± 3.5/59.9 ± 44.9	74.96 ± 5.84	29.2 ± 6.3/59.9 ± 44.9	51.31 ± 10.44	7.8 ± 4.5/59.9 ± 44.9	86.92 ± 7.57

9	M49	25.9 ± 14.8/278.5 ± 251	90.99 ± 5.15	31.6 ± 16.2/278.5 ± 251	89.01 ± 5.64	24.8 ± 22.1/278.5 ± 251	91.39 ± 7.68	22.5 ± 16.3/278.5 ± 251	92.17 ± 5.67
10	M22	52.9 ± 44.4/3437 ± 1099	98.46 ± 1.29	155.4 ± 66.7/3437 ± 1099	95.48 ± 1.94	246.1 ± 66.8/3437 ± 1099	92.84 ± 1.94	24.2 ± 11.6/3437 ± 1099	99.30 ± 0.34
11	ST8	391.3 ± 55.0/2336 ± 381	83.25 ± 2.36	663.2 ± 124.9/2336 ± 381	71.60 ± 5.35	485.8 ± 59.0/2336 ± 381	79.20 ± 2.52	576.8 ± 26.4/2336 ± 381	75.31 ± 1.13
12	M75	125.3 ± 75.1/1661 ± 1014	92.46 ± 4.52	191.2 ± 48.9/1661 ± 1014	88.49 ± 2.94	119 ± 79.1/1661 ± 1014	92.84 ± 4.77	75.9 ± 28.2/1661 ± 1014	95.43 ± 1.70
13	TR2612	394.3 ± 34.4/7776 ± 3043	94.93 ± 0.44	606.8 ± 163.4/7776 ± 3043	92.20 ± 2.10	212.6 ± 21.1/7776 ± 3043	97.27 ± 0.27	440.6 ± 69.2/7776 ± 3043	94.33 ± 0.89
14	ST3	3.0 ± 1.2/14.7 ± 8.3	79.59 ± 7.86	2.0 ± 1.1/14.7 ± 8.3	86.39 ± 7.35	1.1 ± 1.3/14.7 ± 8.3	92.35 ± 8.95	6.0 ± 2.6/14.7 ± 8.3	59.18 ± 17.78
15	ST10	22.2 ± 9.6/1284 ± 961	98.27 ± 0.75	82.6 ± 31.6/1284 ± 961	93.56 ± 2.46	52.6 ± 22.0/1284 ± 961	95.90 ± 1.71	61.8 ± 14.8/1284 ± 961	95.19 ± 1.15
16	ST6735	30.5 ± 4.5/87.5 ± 47	65.14 ± 5.13	75.7 ± 13.0/87.5 ± 47	13.52 ± 14.84	49.5 ± 6.5/87.5 ± 47	43.43 ± 7.43	51.0 ± 7.7/87.5 ± 47	41.71 ± 8.80
17	M13	72.6 ± 31.2/1130 ± 571	93.57 ± 2.76	196.5 ± 27.0/1130 ± 571	82.61 ± 2.39	320.3 ± 124.4/1130 ± 571	71.65 ± 11.0	46.6 ± 25.0/1130 ± 571	95.87 ± 2.21
18	M18	386.0 ± 68.2/2113 ± 650	81.73 ± 3.23	362.1 ± 157.9/2113 ± 650	82.86 ± 7.47	215.3 ± 195.4/2113 ± 650	89.81 ± 9.25	124.2 ± 54.2/2113 ± 650	94.12 ± 2.57
19	M74	270.8 ± 75.2/664.5 ± 228	59.24 ± 11.32	429.8 ± 83.4/664.5 ± 228	35.31 ± 12.55	383 ± 60.6/664.5 ± 228	42.36 ± 9.12	368.5 ± 42.9/664.5 ± 228	44.54 ± 6.45
20	M5	375.6 ± 47.4/1092 ± 154.9	65.60 ± 4.34	608.5 ± 24.2/1092 ± 154.9	44.28 ± 2.21	645.7 ± 54.3/1092 ± 154.9	40.87 ± 4.97	303.2 ± 44.4/1092 ± 154.9	72.23 ± 4.07

* The value is an average of experiment done in quadruplicate.

IMS/NMS: mean CFU in immune serum ± SD/mean CFU in normal serum ± SD

**Table 3:** Bactericidal activity (mean % reduction in CFU) of J14 peptide antisera against Thai GAS isolates with different numbers of M protein C-repeats.

**Isolate no.**	**Number of C-repeat**	**Bactericidal activity (% reduction)**			
		**J14 (KALE)**	**J14.1 (ADLA)**	**J14-R1 (KDLA)**	**J14-R2 (AEHQ)**
1	single	52.89 ± 10.36*	44.91 ± 9.62*	38.43 ± 8.63*	58.67 ± 8.54*
2–8	two	84.87 ± 14.49	88.51 ± 9.41	74.04 ± 20.49	88.73 ± 19.52
9–20	three	83.60 ± 13.73	72.94 ± 26.88	77.49 ± 22.44	79.95 ± 21.07

* Significantly less (*P *< 0.05) compared to isolate numbers 2–8 and 9–20.
